# Constitution and By-Laws of the Medical Association of Central New York

**Published:** 1872-08

**Authors:** 


*Constitution, By-Laws, Officers and Permanent Members, of the Medical Association of Central New York.*

From Article 3 of the constitution, we learn that this Association is composed of delegates from the Medical Societies of the counties of Onondaga, Cayuga, Seneca, Ontario, Wayne and Monroe. Any other County Society by conforming to the constitution and electing delegates, is entitled to admisssion. The meetings which are to occur in June and December, are held alternately in Rochester or Syracuse.

We had the pleasure of attending the last Meeting of this Society, held in Rochester in June last, and were highly pleased at the zeal and intelligence on medical subjects there displayed. Many of the papers which were presented to the Association were of much interest, and we hope ere long to present some of them to our readers,

The address of the president, Dr. B. L. Hovey of Rochester, on "Thoughts and Reflections for the People," was a thoughtful and interesting paper, and was listened to with respectful and appreciative attention. We wish for this society a long life and many pleasant meetings, and are under many obligations to its officers and members for their kind attention paid to us at their late meeting at Rochester. The officers for the ensuing year are as follows: Dr. S. Gilmore, President; Dr. A. Bolter, 1st Vice President; Dr. H. N. Eastman, 2d Vice President; Dr. T. S. Brinkerhoff, of Auburn, Secretary; Dr. A. Mercer of Syracuse, Treasurer.

At this meeting a large delegation was present, the following counties being represented: Cayuga, Chemung, Cortland, Erie, Genesee, Livingston, Monroe, Ontario, Onondaga, Otsego, Seneca, Wayne, and Yates.

